# Supplementary material for: Genomic Biomarkers of Survival in Patients with Adenocarcinoma of the Uterine Cervix Receiving Chemoradiotherapy
Source: Int J Mol Sci. 2020 Jun 9;21(11):4117. doi: 10.3390/ijms21114117 (PMC7312424; doi:10.3390/ijms21114117)
Supplement: Supplementary file 1 [file ijms-21-04117-s001.pdf]

Supplementary Materials

Table S1. Nonsynonymous mutations of the individual tumors.

Patient	Tumor mutation number	mutation 1	mutation 2	mutation 3	mutation 4	mutation 5	mutation 6	mutation 7	mutation 8	mutation 9	mutation 10
Cancer DNA sequencing with target cancer panel											
1	4	TP53	POLD1	SF3B1	ATRX						
2	71	TP53	FGFR2*	KMT2A*	NOTCH2	PIK3CA*	ATM	NF1	PIK3R1	ARID1B	BRAF
3	6	PTEN	TSC2	BLM	PIK3R1*	SETBP1	ECORL1				
4	27	TP53	FGFR2*	PIK3R1*	NOTCH1	NOTCH2	ATR	RET*	POLI	ARIDIA	AR*
5	5	SMAD4	BRIP1	SMRCA4	KMT2C						
Whole exome sequencing plus cancer DNA sequencing with target cancer panel											
6	8	GEN1	GNAS*	CDKN2C	SMARCA4	PPM1D	GRIN2A	CIC	IDH1*		
7	7	AR*	ARID1A	KAT6A	PBRM1	EP300	HNF1A	RAD1			
8	5	ATR	KMT2D	BCORL1	PLCG1						
9	12	ARID1A	TP53	PTEN	ERBB2*	CUX1	KMT2D	RAD50	STAG2	SETD2	STK11
10	8	FLT4*	POLE	RB1	TSC2	AXIN1	EXO1	KMT2D	RITI		
11	2	TSC1	BRCA2								
12	13	ARID1A	ATM	PTEN	PIK3CA*	ARID1B	FBXW7	TET2	APC	RAD50	NRAS*
13	3	PIK3CA*	KDR	TCF3							
14	3	GNAQ*	LRP1B	KDM5C							
15	3	PIK3CA*	LRP1B	CUX1							
16	5	AR*	ATM	BRIP1	MET*	KMT2D					
17	2	DDR2	SMC1A								
18	5	TP53	XRCC2	KMT2D	SMAD4	TGFBR2					
19	7	ATR	PLCG1	KMT2C	PTEN	PAK3	ROS1*	RHOA			
20	6	IGF1R*	NOTCH3	TCF3	ARID2	FBXW7	SDHB				
21	4	AR*	CSF1R*	NOTCH3	NOTCH1						

Note: 1. Somatic mutation numbers in patient 2, 4, 9, and 12 were over 10. Thus, complete information on the mutated genes in these patients cannot be provided in this table.

2. Asterisk represents mutations of oncogenes.

Table S2. Predictive immunohistochemical and clinical parameters, tumor mutation number, and AUC.

**Variables**	**Disease-relapse AUC / *p* value**	**Local failure AUC / *p* value**	**Distant metastasis AUC / *p* value**
Mcl-1 H-score	0.66 ± 0.12/0.23	0.51 ± 0.13/0.94	0.54± 0.16/0.80
c-Myc H-score	0.49 ± 0.13/0.40	0.61 ± 0.12/0.42	0.18 ± 0.11/0.035*
IGF H-score	0.61 ± 0.13/0.42	0.63 ± 0.13/0.34	0.53 ± 0.16/0.89
PD-L1 combined positive score	0.49 ± 0.13/0.94	0.65 ± 0.12/0.24	0.19 ± 0.09/0.043*
TNF-α H-score	0.16 ± 0.09/0.007*	0.31 ± 0.11/0.16	0.28 ± 0.12/0.13
Maximum tumor dimension	0.60 ± 0.13/0.46	0.57 ± 0.14/0.61	0.71 ± 0.13/0.17
Pretreatment hemoglobulin	0.41 ± 0.13/0.24	0.48 ± 0.14/0.39	0.38 ± 0.19/0.41
Pretreatment serum CEA	0.41 ± 0.13/0.48	0.51 ± 0.14/0.91	0.59 ± 0.17/0.54
Tumor mutation number	0.26 ± 0.11/0.06	0.20 ± 0.10/0.023	0.49 ± 0.15/0.93

Abbreviations: AUC = area under the receiving operating characteristic curve.
CEA = carcinoembryonic antigen.

A **B**

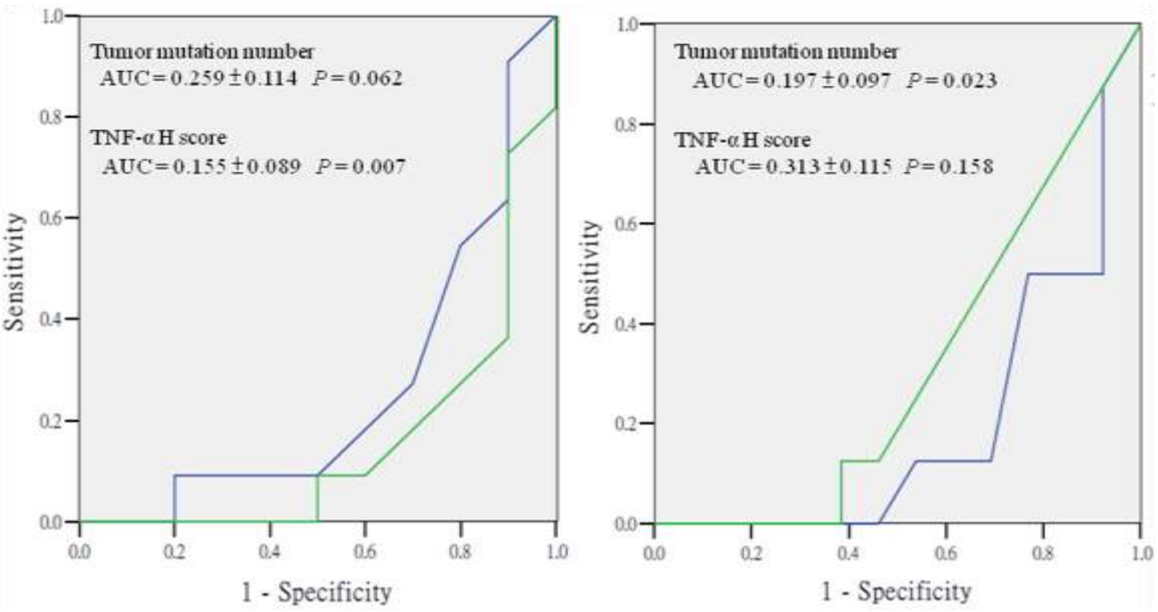

Figure S1. Area under the receiver operating characteristic curve (AUC) for the tumor mutation number (TMN) and the *TNF-α* H score for patients with disease relapse (**A**), and local recurrence (**B**).

Table S3. MCL1 amplification, tumor mutation number, and treatment outcomes.

Patients	Local recurrence	Distant metastasis	Cancer death	Disease relapse	MCL1 amplification	Tumor mutation number
DNA sequencing in target cancer panel						
1		+	+	+		4
2						71
3		+		+	+	6
4					+	27
5						5
Whole exome sequencing plus DNA sequencing in target cancer panel						
1						8
2	+	+	+	+	+	7
3	+			+		5
4						12
5						8
6	+	+		+	+	2
7		+	+	+	+	13
8	+			+		3
9	+			+		3
10	+			+	+	3
11	+			+	+	5
12						2
13	+			+		5
14						7
15						6
16						4

Note: "+" means a positive event.

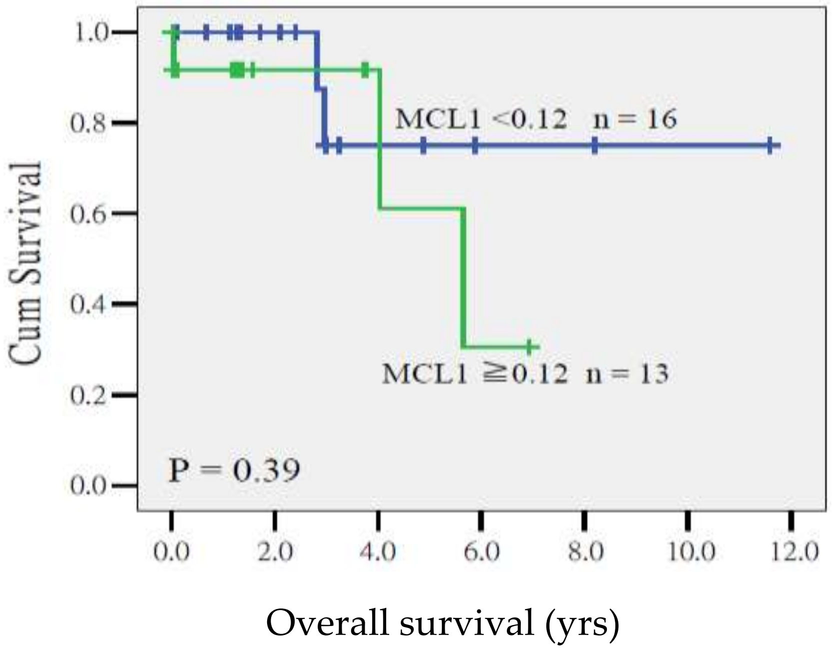

Figure S2. External validation in TCGA. Overall survival of the 29 samples in TCGA was stratified by those with or without MCL1 amplification using a median value of the copy number. Although certain patients with MCL1 amplification were likely to have inferior overall survival compared with those without amplification, there was no statistically significant difference between the two groups (p = 0.39).

Cum Survival: Cumulative Survival

Note: 1. Median follow-up duration was 2 years.

2. Five patients died, three in the amplification-positive and two in amplification-negative groups.

A

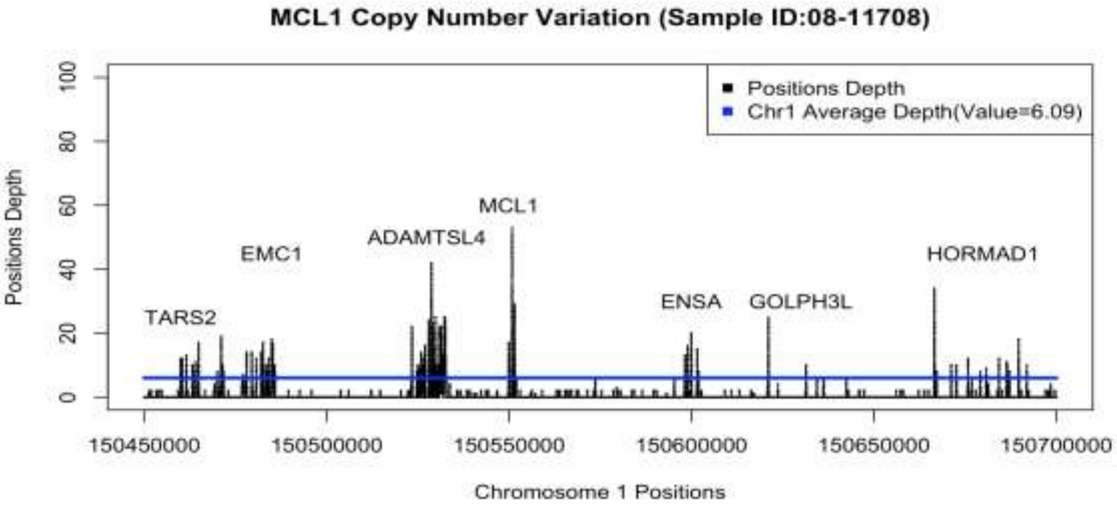

B.

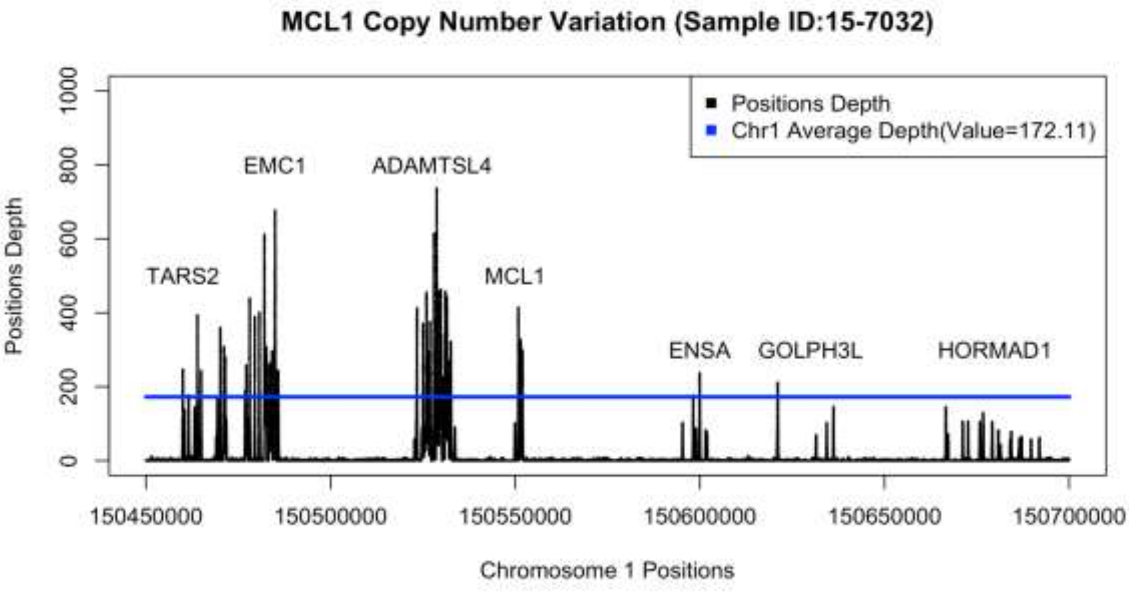

Figure S3. Example of tumors with MCL1 amplification (**A**) and without MCL1 amplification (**B**). Note: Samtools was used to obtain the sequencing depth of each point on chromosome 1 from the bam file, and the average depth of chromosome 1 as standard copy was calculated. The sequencing depth of MCL1 was compared with six genes near MCL1 to determine the copy number variation of the MCL1 gene.
